# *Patient Safety in Surgery* – announcing the journal’s first impact factor (3.7)

**DOI:** 10.1186/s13037-023-00375-8

**Published:** 2023-08-30

**Authors:** Philip F. Stahel, Sebastian Weckbach, Navid Ziran, Wade R. Smith, Ernest E. Moore, Hans-Christoph Pape, Pierre-Alain Clavien

**Affiliations:** 1 Mission Health, 50 Schenck Pkwy, Asheville, NC 28803 USA; 2 Department of Surgery, Brody School of Medicine, East Carolina University, E. 5th St., Greenville, NC 27858 USA; 3 Department of Specialty Medicine, College of Osteopathic Medicine, Rocky Vista University, Parker, CO USA; 4 NeuroSpine Zürich, Zurich, Switzerland; 5 Department of Orthopedics, St. Joseph’s Medical Center, Phoenix, AZ USA; 6 Department of Orthopedics, Swedish Medical Center, Englewood, CO USA; 7 Ernest E. Moore Shock Trauma Center, Denver Health, Denver, CO USA; 8 Department of Trauma Surgery, University Hospital Zurich, Zurich, Switzerland; 9 Department of Surgery and Transplantation, University Hospital Zurich, Zurich, Switzerland

**Keywords:** Patient safety in surgery, Impact factor, Publication metrics, Journal citation reports

## A pioneer journal in the field of surgical patient safety

**Fig. 1:**
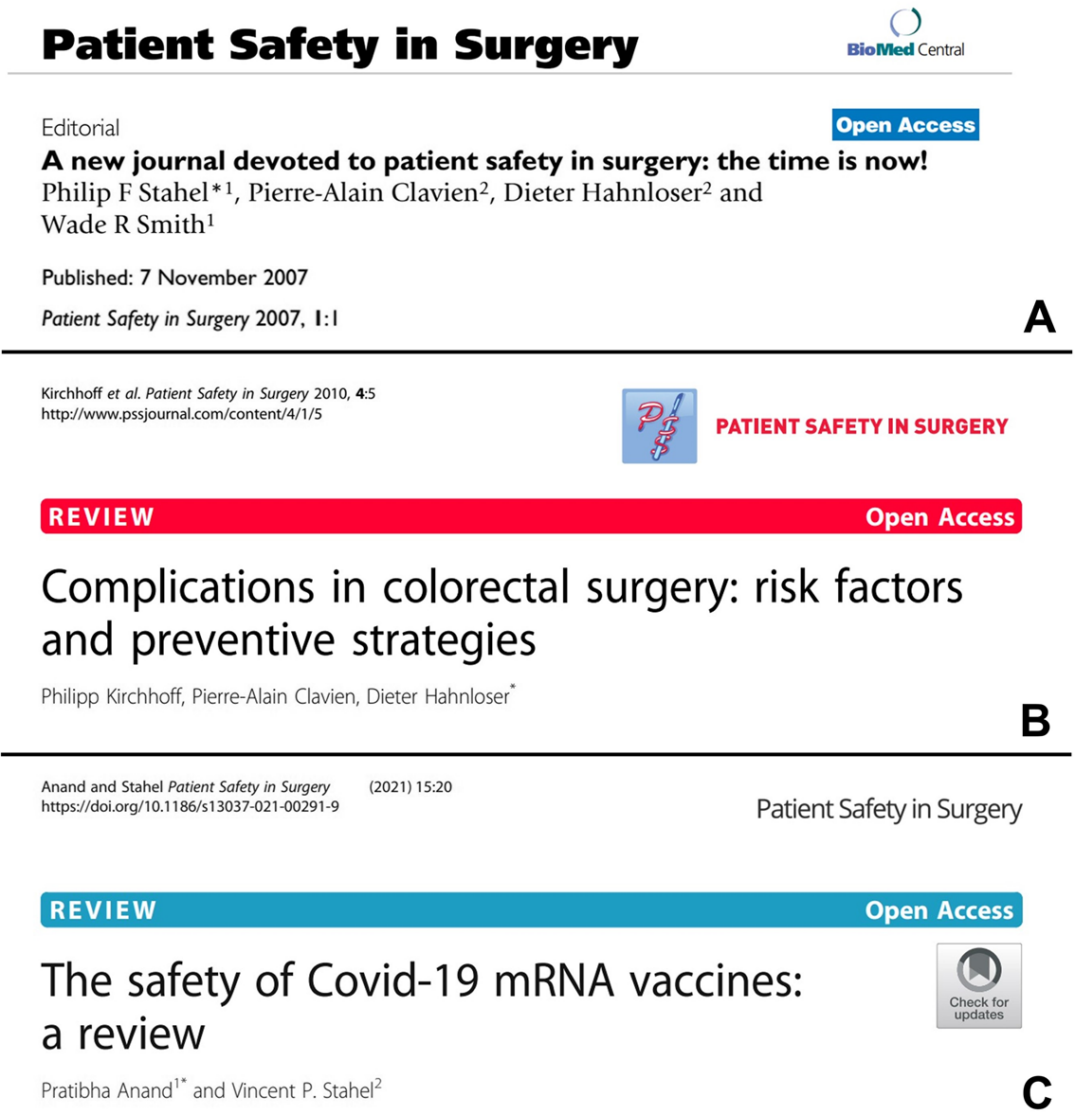
Evolution of the journal’s layout design over the past 15 years. **A** *Patient Safety in Surgery* launch editorial, published on November 7, 2007. **B** The journal’s highest accessed and most cited article of all times, with 234,000 accesses and 231 citations at the time of publication of this editorial. **C** The journal’s fastest high-citation publication of all times, with 216,000 accesses and 100 citations within two years of publication (May 1, 2021). This article was the #1 driver of the journal’s first official impact factor (3.7) on June 28, 2023. (Source: www.pssjournal.com)

**Fig. 2:**
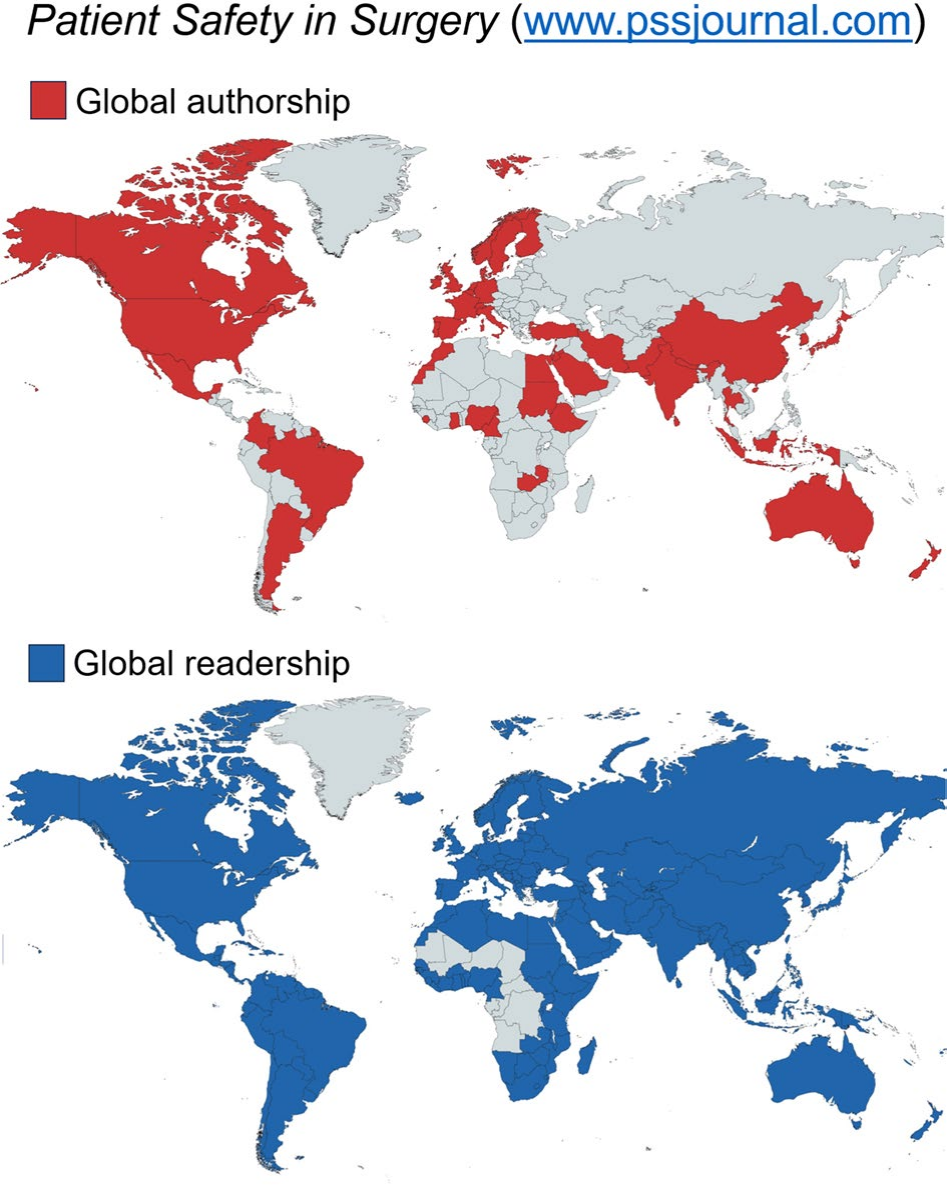
The journal’s global authorship (upper panel) and readership (lower panel), as of July 5, 2023. (Source: www.pssjournal.com)

*Patient Safety in Surgery* was launched as an independent, open-access, peer-reviewed and PubMed-indexed journal on November 7, 2007 (Fig. 1A) [1]. The journal’s global visibility has incrementally increased since its inception almost 16 years ago (Fig. 2). Authors from more than 50 countries and around 300 different institutions have published in the journal until present. While the first edition in 2007 had less than 2,000 articles accessed per month, this metric increased to more than 250,000 monthly article accesses at the time of the journal’s 10-year anniversary in 2017 [2]. The single highest impact article in the journal (at the time of writing this editorial) showed 234,000 accesses and 231 citations since its publication in March 2010 (Fig. 1B) [3]. The journal’s editorial board is consistently scrutinizing the scientific quality of submitted articles by rejecting a majority of poor-quality submissions before peer review. This stringent editorial process is intended to decrease the burden of the inflated number of reviewing requests to our referees [4].

**Fig. 3:**
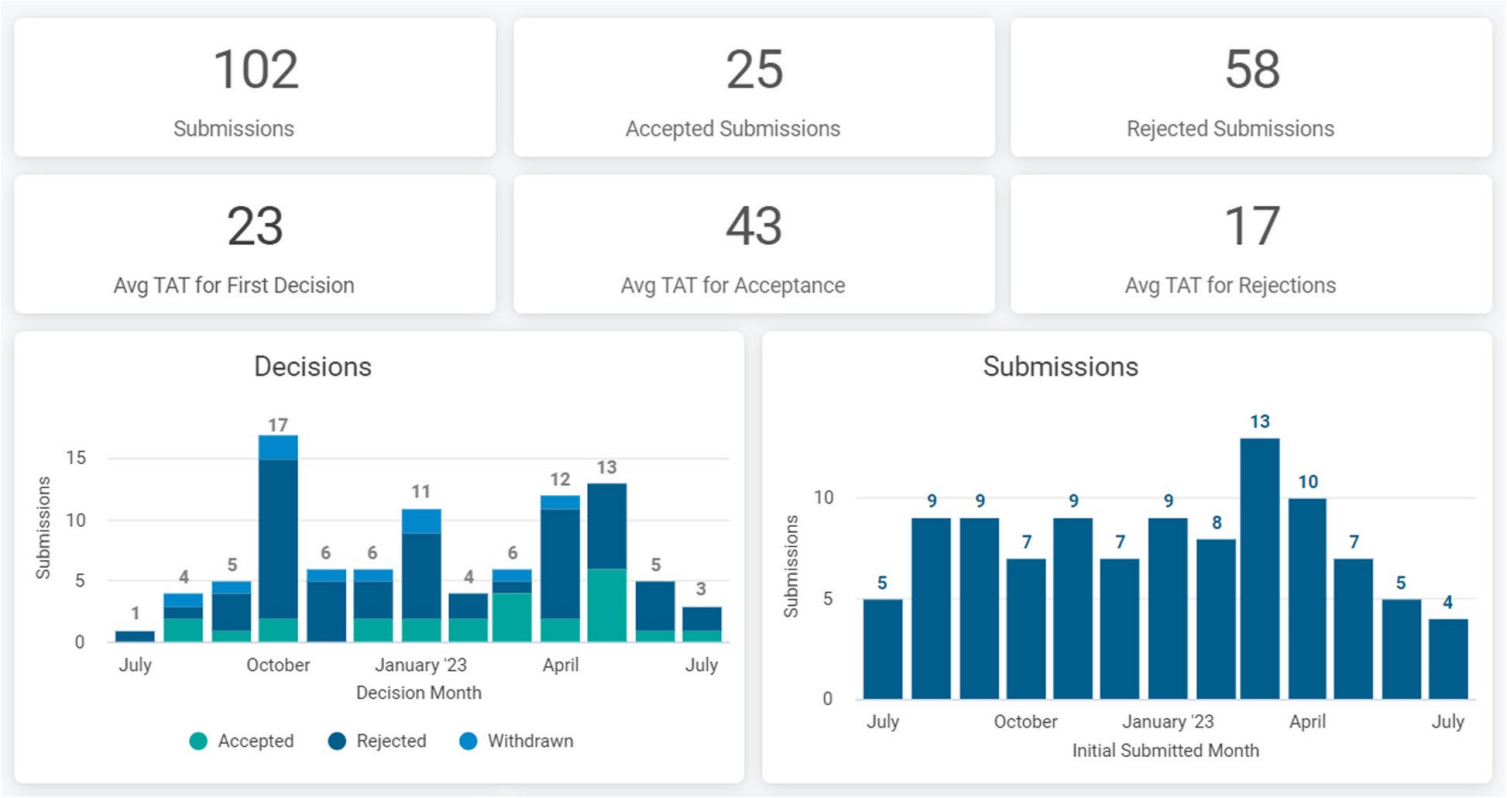
Journal publication metrics over the past rolling 12 months, as of July 5, 2023. Abbreviation: TAT, turnaround time. (Source: www.pssjournal.com)

Owing to the high benchmark for article acceptance, *Patient Safety in Surgery* has gradually increased the journal’s rejection rate from 20% (2012), to 50% (2020), and currently 70% (2023). Since many articles are rejected at the editorial board screening stage, the average time to rejection is as short as 17 days. Articles submitted for peer review have an average turnaround (TAT) time of 23 days until a first decision is made, and 43 days until acceptance for publication (Fig. 3). In spite of the successful academic and operational metrics, the journal has been lacking an official impact factor to allow benchmarking its scientific renown among the currently more than 20,000 peer-reviewed scientific journals available on the market.

## The first official impact factor

On occasion of the journal’s 5^th^ anniversary in 2012, we published an editorial that included the following historic statement [5]:

**Table:** 

*“The first international editorial board meeting for Patient Safety in Surgery took place on October 3, 2012, at the Clinical Congress of the American College of Surgeons (ACS) in Chicago, IL. During this meeting, we discussed current challenges and outlined the future vision for the journal in the next 5 years. One priority is to take the journal from its first unofficial impact factor of 1.19 to an official impact factor in the Journal Citation Reports.”*	

**Fig. 4:**
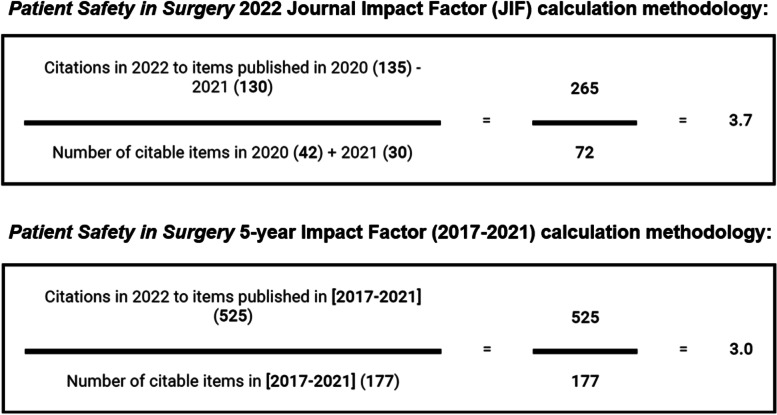
Calculation methodology for the journal’s 2022 impact factor (upper panel) and 5-year impact factor (lower panel). (Source: Journal Citation Reports™ © Clarivate 2023)

On June 28, 2023, after a wait time of almost 16 years since November 7, 2007, *Patient Safety in Surgery* received the first Journal Impact Factor (JIF) of 3.7 by Clarivate Journal Citation Reports™. The JIF is calculated as the number of citations of articles published in the journal during the preceding two years (numerator) divided by the number of citable articles in the journal during the same time-window (denominator). The citable items in the denominator are reflective of the scholarly contributions of the journal, including original research, case reports, and review articles. In contrast, editorials and letters which serve a different communication role outside of scholarly activity are excluded from the denominator. For calculation of the current impact factor for *Patient Safety in Surgery*, articles published in 2020 and 2021 were cited 265 times in 2022, divided by a total of 72 citable articles published in 2020 and 2021, resulting in a respectable impact factor of 3.7. While the time-window of the two preceding years defines the standard impact factor, some journals with a slower velocity of reaching citation peaks prefer to leverage the 5-year impact factor, which is reflective of the years 2017–2021 for the 2022 JIF (Fig. 4). The new JIF for *Patient Safety in Surgery* implies that an average article published in the journal has been cited about 3.7 times within two years after publication. The top-5 most cited articles which contributed to the new JIF for *Patient Safety in Surgery* are listed in Table 1. Notably, these citations are exclusively reflective of review articles which are preferentially cited in other publications. Impressively, 68 of 72 citable articles in 2020–2021 were cited at least once, extrapolating to a citation rate of 94% for the journal. Only four original papers published in the journal during 2020 and 2021 were not cited in 2022. The stratification of the 68 cited articles by publication type, specialty area, and country of origin is shown in Table 2. The most cited article with 172 citations was an editorial entitled *“How to risk-stratify elective surgery during the COVID-19 pandemic?”* which was published shortly after the World Health Organization (WHO) declared the novel coronavirus disease 2019 (COVID-19) as a global pandemic in March 2020 [6]. However, since editorials represent non-citable items, this article was not included for the 2022 JIF tracking (as this current editorial will be excluded as well). The #1 ranking citable article published during the 2020–2021 JIF time-window was cited 58 times in 2022, after its publication on May 1, 2021, and 100 times overall until present [7]. This review article was published around the peak of the global controversy around the safety and efficacy of the new generation mRNA vaccines during the COVID-19 pandemic (Fig. 1C).

**Table 1:** Top-5 most cited articles that contributed to the journal’s 2022 impact factor. (Source: Journal Citation Reports™ © Clarivate 2023)

**Rank**	**Reference**	**Article Type**	**Year**	**Country**	**Citations**
1	Stahel VP, Anand P. The safety of COVID-19 mRNA vaccines: a review. *Patient Saf. Surg.* 2021, 15:20	Review	2021	USA	58
2	Abate SM, et al*.* Postoperative mortality among surgical patients with COVID-19: a systematic review and meta-analysis. *Patient Saf. Surg.* 2020, 14:37	Review	2020	Ethiopia	14
3	Gedefaw G, et al*.* Prevalence, indications, and outcomes of Caesarean section deliveries in Ethiopia: a systematic review and meta-analysis. *Patient Saf. Surg.* 2020, 14:11	Review	2020	Ethiopia	10
4	Brown NJ, et al*.* Ethical considerations and patient safety concerns for cancelling non-urgent surgeries during the COVID-19 pandemic: a review. *Patient Saf. Surg.* 2021, 15:19	Review	2021	USA	9
5	Weprin S, et al*.* Risk factors and preventive strategies for unintentionally retained surgical sharps: a systematic review. *Patient Saf. Surg.* 2021, 15:24	Review	2021	USA	8

**Table 2:** Underlying metrics of the 68 cited articles that contributed to the journal’s 2022 impact factor

	**No. of cited articles published in 2020**^a^	**No. of cited articles published in 2021**^a^	**No. (%) of cited articles published in 2020-2021**^a^
***Article type***			
Original research	25	21	**46 (67%)**
Review article	3	5	**8 (12%)**
Systematic review	4	2	**6 (9%)**
Case report	5	1	**6 (9%)**
Hypothesis article	2	0	**2 (3%)**
***Specialty area***			
Operating room safety	11	7	**18 (26%)**
COVID-19	8	5	**13 (19%)**
Orthopedic Surgery	7	5	**12 (17%)**
Trauma Surgery	6	2	**8 (11%)**
Colorectal Surgery	1	4	**5 (7%)**
Obstetrics	3	0	**3 (4%)**
Anesthesia	2	1	**3 (4%)**
Intensive Care	0	1	**1 (1.5%)**
Hepatobiliary Surgery	1	0	**1 (1.5%)**
Bariatric Surgery	1	0	**1 (1.5%)**
Plastic Surgery	1	0	**1 (1.5%)**
Oncological Surgery	0	1	**1 (1.5%)**
Spine Surgery	0	1	**1 (1.5%)**
Urology	0	1	**1 (1.5%)**
Dentistry	0	1	**1 (1.5%)**
***Country of origin***			
USA	13	12	**25 (37%)**
Ethiopia	6	1	**7 (10%)**
Japan	3	1	**4 (6%)**
India	1	2	**3 (4%)**
Brazil	2	1	**3 (4%)**
Germany	1	2	**3 (4%)**
Switzerland	2	0	**2 (3%)**
Netherlands	2	0	**2 (3%)**
United Kingdom	0	2	**2 (3%)**
Sweden	0	2	**2 (3%)**
Australia	0	2	**2 (3%)**
Iran	1	1	**2 (3%)**
Jordan	1	1	**2 (3%)**
Lebanon	1	0	**1 (1.5%)**
Egypt	1	0	**1 (1.5%)**
Morocco	1	0	**1 (1.5%)**
Saudi Arabia	1	0	**1 (1.5%)**
China	0	1	**1 (1.5%)**
Thailand	0	1	**1 (1.5%)**
South Korea	1	0	**1 (1.5%)**
Finland	1	0	**1 (1.5%)**
Italy	1	0	**1 (1.5%)**

^a^Articles are listed if they were cited at least once in 2022. The number of individual *articles* does not match the number of overall *citations* shown in Fig. 4

## The journal’s future vision

In spite of the editorial board’s excitement about the journal’s first impact factor, we remain cognizant that the JIF represents a measure of the global impact of *Patient Safety in Surgery* and is not necessarily a testament to the scientific quality of the research published in the journal. Therefore, our editorial board is intentionally not promoting the JIF in isolation, but rather alongside a multiplicity of additional publication metrics, as outlined above.

We remain committed to upholding the highest level of scrutiny and adherence to our established peer review process for assurance of high-quality science published in the journal. We would like to thank our readers, authors, reviewers, and editorial board members for their unwavering support of the journal’s mission over the past 16 years: to improve the safety of patients undergoing surgical procedures, on a global scale.

## Data Availability

Please contact the authors for data requests.
